# Cerebral arterial vascularization of the scimitar-horned oryx (*Oryx dammah*)

**DOI:** 10.1007/s11259-023-10253-4

**Published:** 2023-11-09

**Authors:** Maciej Zdun, Jakub Jędrzej Ruszkowski, Jarosław Sobolewski, Maciej Gogulski

**Affiliations:** 1 Department of Basic and Preclinical Sciences, Nicolaus Copernicus University in Torun, Lwowska 1, Torun, 87-100 Poland; 2 Department of Animal Anatomy, Poznan University of Life Sciences, Wojska Polskiego 71C, Poznan, 60-625 Poland; 3 University Centre for Veterinary Medicine, Szydłowska 43, Poznan, 60-656 Poland; 4 Department of Public Health Protection and Animal Welfare, Institute of Veterinary Medicine, Faculty of Biological and Veterinary Sciences, Nicolaus Copernicus University, ul. Lwowska 1, Toruń, 87-100 Poland; 5 Department of Preclinical Sciences and Infectious Diseases, Poznan University of Life Sciences, Wołynska 35, Poznan, 60- 637 Poland

**Keywords:** Arterial circle of the brain, Artery, Neuroanatomy, Oryx

## Abstract

The Scimitar-horned Oryx (*Oryx dammah)* is a large terrestrial mammal native to Africa. Since the year 2000, it is classified as extinct in the wild. It is a subject of various conservation projects. The aim of this study was to describe the arterial vascularization of the brain in this species of oryx. Three different anatomical methods were used to obtain a complete arterial pattern - latex injection, corrosion cast, and computed tomography. The arterial vascularization of the brain was described. The main components of the cerebral arterial circle were the rostral cerebral arteries and the caudal communicating arteries. These vessels were created from the intracranial part of the internal carotid artery, that emerged from the rostral epidural rete mirabile. In the juvenile specimen, the whole internal carotid artery was observed. The anatomical pathways of the blood supply to the brain are important during medical procedures in cases of congestion and fainting caused by inadequate brain blood perfusion.

## Introduction

Scimitar-horned Oryx (*Oryx dammah)* is a large, terrestrial mammal native to Africa. From the year 2000, it was classified by IUCN as extinct in the wild with identified conservation sites. It is bred in captivity in a special reserves. Different methods used can create a complete image of the vascular pattern of the described area with topographic relations to soft tissue and the skull. Such detailed descriptions can be used later in establishing veterinary protocols for physiological and clinical studies. The anatomical pathways of the blood supply to the brain are important during medical procedures in cases of congestion and fainting caused by inadequate brain blood perfusion (Kid et al. 2016).

Anatomical descriptions like the one in this study may contribute to further research in the field of physiology and evolutionary sciences. The area described has been the subject of studies concerning anatomical and physiological adaptations to the environment, due to the morphological and functional characteristics of the structure of the rostral epidural rete mirabile (Graczyk and Zdun 2021). This is the first description of the anatomy of the arterial vessels of the brain in Scimitar-horned Oryx. The aim of this study is to describe the anatomy of the arterial vessel of the brain in Scimitar-horned Oryx.

## Materials and methods

### Animals

The study was conducted on cadavers of 3 adults and 1 juvenile—7 months old (2 males, 2 females) Scimitar-horned Oryx obtained from zoos. The animals were delivered as post-mortem specimens. Only animals without head or neck trauma, neurological or cardiovascular diseases were included in the study. No animal was killed in order to perform this study.

## Methods

Three methods were used to obtain detailed patterns of arterial vessels supplying blood to the brain. The methods were used on dissected heads with necks.

The first method used on one specimen was injecting bilateral common carotid arteries with colored liquid LBS 3060 latex. After the injection, the preparation was submerged in 5% formaldehyde solution and then manually prepared to obtain blood vessels on soft tissue.

The second method used on two specimens was Duracryl® Plus introduced to bilateral common carotid arteries. After the injection, the specimens were acerated enzymatically (Persil®) at a temperature of 40 °C for 30 days. This method resulted in obtaining a corrosion cast of arteries on the bone scaffold.

The third method used on one cadaver (juvrnile animal) was the contrast-enhanced cone-bean computed tomography scan (Fidex, Animage, USA). Prior to the scan, bilateral common carotid arteries were injected with a contrast agent (barium sulphate; barium sulphuricum 1.0 g/mL, Medana®, Poland). The scans were performed at the University Centre for Veterinary Medicine in Poznan, Poland, with scanning parameters of 110 kVp, 0.08 mAs pet shot, 20.5 mAs (Total mAs). The scan was post-produced in FidexGUI (version 3.6.0, Animage, USA) and 3D Slicer software (ver. 5.40) This way, a maximum intensity projection reconstruction and 3D model of the vascular pattern were obtained.

## Results

**Fig. 1:**
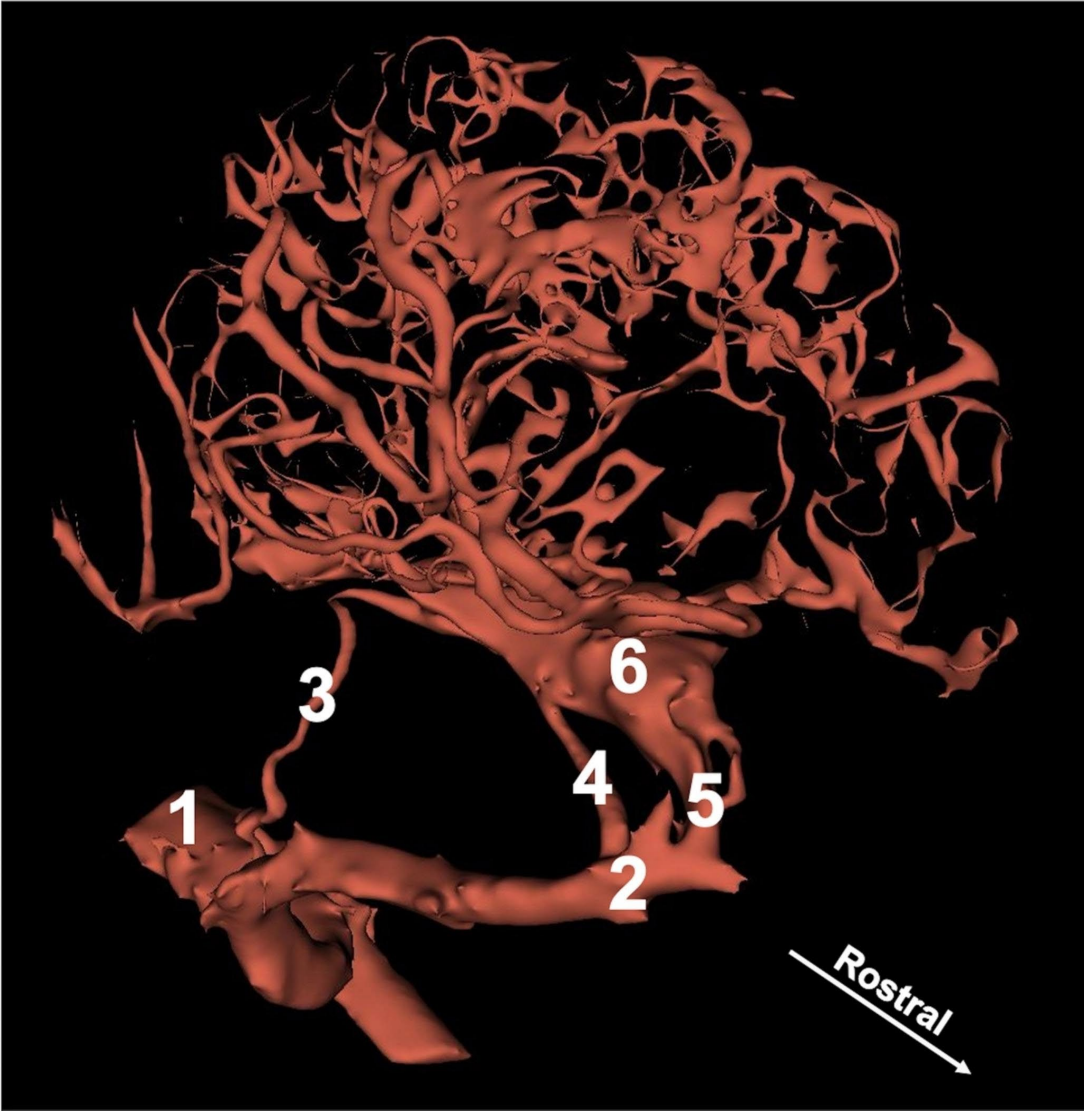
3D reconstruction of the arterial vessel supplying blood to the brain of the Scimitar-horned Oryx. The reconstruction was based on the cone-beam computed tomography angiography scan. 1—common carotid artery. 2—maxillary artery. 3—internal carotid artery. 4—caudal branch to the rostral epidural rete mirabile. 5—rostral branches to the rostral epidural rete mirabile. 6—rostral epidural rete mirabile

The main arterial vessel supplying the head with blood is the common carotid artery. At the level of the occipital condyle, the internal carotid artery branched off (Fig. 1).

**Fig. 2:**
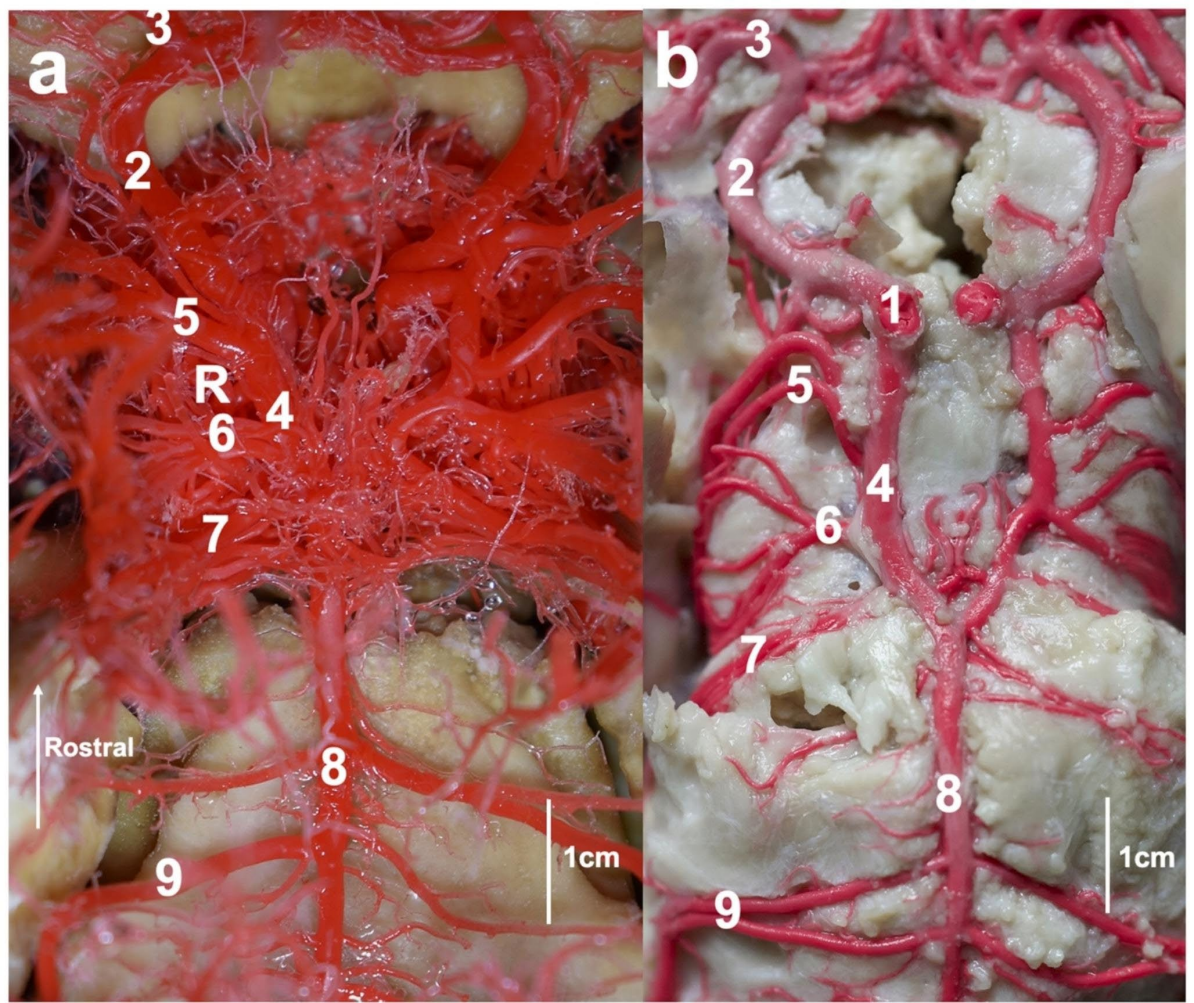
Cerebral arterial circle: a—corrosion cast, dorsal view; b—latex preparation, ventral view. R—rostral epidural retes mirabiles (under the cerebral arterial circle). 1—intracranial part of the internal carotid artery. 2—rostral cerebral artery. 3—middle cerebral artery. 4—caudal communicating artery. 5—caudal cerebral artery. 6—caudal choroidal artery. 7—rostral cerebellar artery. 8—basilar artery. 9—caudal cerebellar artery

**Fig. 3:**
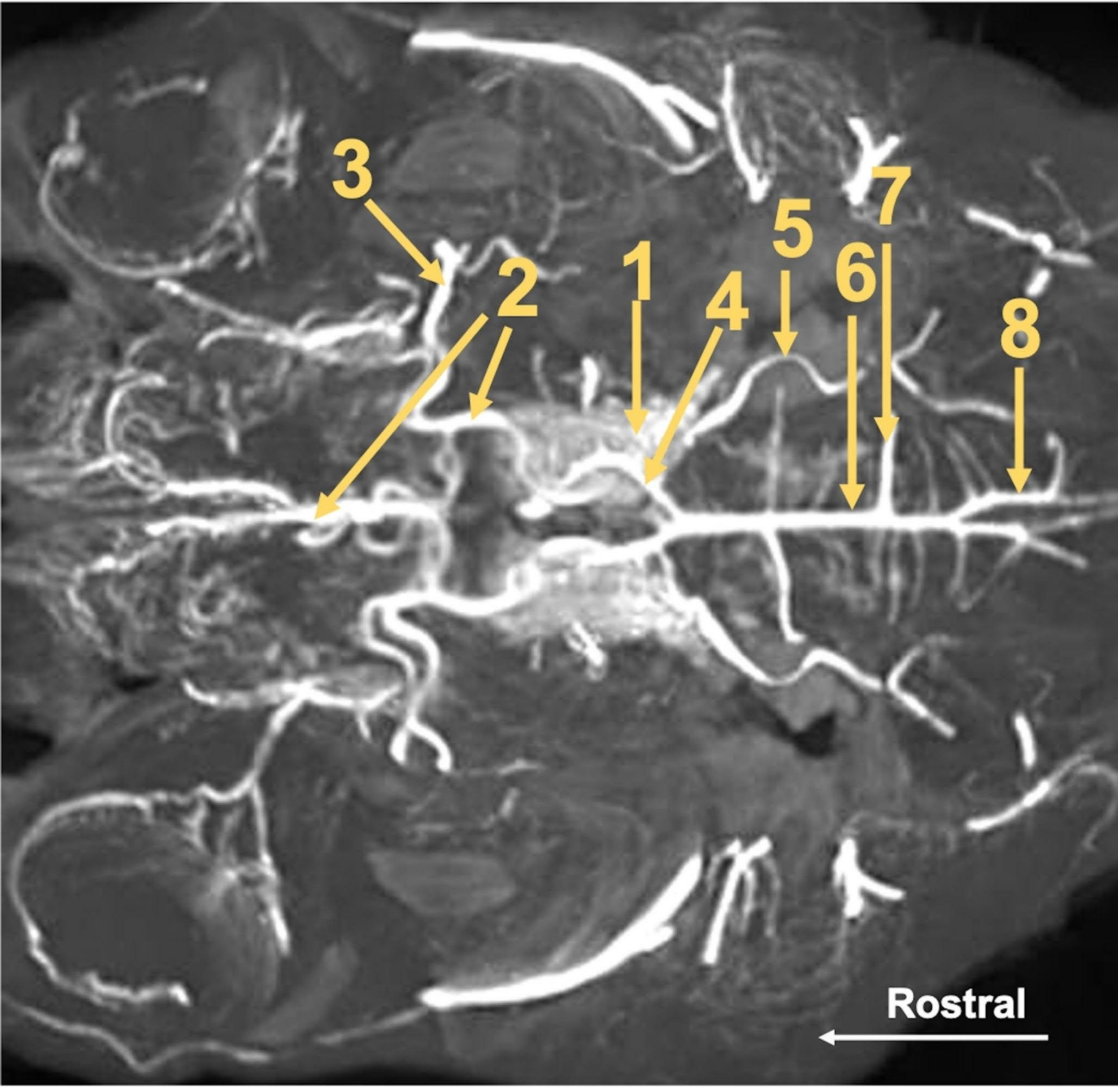
Maximum intensity projection reconstruction of the CT scan of the arterial circle of the brain and its branches in Scimitar-horned Oryx. 1—rostral epidural rete mirabile. 2—rostral cerebral artery. 3—middle cerebral artery. 4—caudal communicating artery. 5—rostral cerebellar artery. 6—basilar artery. 7—caudal cerebellar artery. 8—ramus spinalis of the vertebral artery

At the initial part of this vessel, a slight thickening is observed. It is a carotid sinus. The internal carotid artery enters the cranial cavity via the jugular foramen and contributes to the rostral epidural rete mirabile from its caudal side. This vessel is present in the juvenile specimens. In adults, the extracranial part of this artery is not observed. When the internal carotid artery diverges, the main artery of the head is called the external carotid artery. Rostrally, this vessel becomes the maxillary artery. From this artery, the caudal branch to the rostral epidural rete mirabile branches off. This is a single, relatively strong vessel passing through the oval foramen. Next, the rostral branches to the rostral epidural rete mirabile divide from the maxillary artery. These vessels join the rostral epidural rete mirabile from its rostral side, entering the cranial cavity through the orbitorotundum foramen. These vessels are present in numbers of 2–4. The rostral epidural rete mirabile is located at the bottom of the cranial cavity. Is a paired, well-developed structure. Each side structure is connected with the one secondary by a few vessels. This makes the shape of bilateral retes look like the letter H. On the cross-section of the rete mirabile, the vessels that compose it have a similar diameter. The rostral epidural rete mirabile creates the intracranial part of the internal carotid artery (mean 3.55 mm). This is a short vessel, which divides into main components of the cerebral arterial circle i.e., the rostral cerebral artery (mean 2.43 mm) and the caudal communicating artery (mean 2.33 mm) (Figs. 2 and 3).

These bilateral vessels form the arterial circle in a shape similar to the heart. The rostral cerebral artery creates the rostrolateral part of the cerebral arterial circle. Three main parts of this vessel can be identified. The first one is from the internal carotid artery to the middle cerebral artery (mean 2.34 mm). It is an arched section that constructs a circle from its lateral side. At the end of this section, the middle cerebral artery branched off. This is a well-developed vessel that provides blood to the largest area of the encephalon. It surrounds the piriform lobe from the anterior side and is arranged on the dorsolateral surface. This artery is divided into several branches, which supply the frontal, parietal, and temporal lobes. The second part of the rostral cerebral artery creates the anterior part of the circle. This part is oriented between the middle cerebral artery and the sagittal plane, where the secondary vessel of the same name is near. The last part of this artery begins where the vessel changes the course and follows in the longitudinal fissure. At the beginning of this part, the rostral communicating artery joins bilateral rostral cerebral arteries. The presence of this artery makes the cerebral arterial circle closed from its anterior side. The last part of the rostral cerebral artery is located on the medial surface of the hemisphere.

The caudal communicating artery creates the caudolateral part of the cerebral arterial circle. This vessel is arcuate. Bilateral arteries assume the shape of the letter U. The first vessel that branched off is the rostral choroidal artery. This is a single vessel. In two specimens, the rostral choroidal artery branched off from the rostral cerebral artery. Next, the caudal cerebral artery (mean 1.25 mm) branched off. In one case, unilaterally, it is a double vessel. This artery supplies the caudal part of the brain.

Further branching off the vessel is the caudal choroidal artery (mean 1.27 mm). It is a single vessel, that is divided into several branches and lies on the mesencephalon. The most posteriorly located branch from the caudal communicating artery is the rostral cerebellar artery (mean 1.28 mm). This vessel lies on the anterolateral surface of the cerebellum.

The basilar artery (mean 1.96 mm) joins the caudal communicating arteries. This is a single vessel, which becomes slightly thinner in the caudal part. From this artery, the caudal cerebellar artery (mean 0.9 mm) branched off. This vessel lies at the caudolateral part of the cerebellum. In one case, this vessel branched off as two vessels, which unified within a few millimeters from the initial point. Such a model of vessels branching off, is present bilaterally. In three cases, this artery branches off asymmetrically. In the initial part of the vessel always departed at a 90-degree angle from the basilar artery. Only in the further part, the vessel headed more caudally. Furthermore, from the basilar artery, the branches to the pons branched off. The mean diameters of the largest vessels supplying blood in arterial blood are shown in Table 1.

**Table 1:** The mean diameters of the largest vessels supplying the brain with arterial blood in Scimitar-horned Oryx

Name of the artery	Mean diameter of the vessel
intracranial part of the internal carotid artery	3.55 mm
rostral cerebral artery	2.43 mm
middle cerebral artery	2.34 mm
caudal communicating artery	2.33 mm
caudal cerebral artery	1.25 mm
caudal choroidal artery	1.27 mm
rostral cerebellar artery	1.28 mm
basilar artery	1.96 mm
caudal cerebellar artery	0.9 mm

## Discussion

This research demonstrated, that in juvenile specimen, the whole, including the extracranial part, the internal carotid artery was present. In adults, the extracranial part was not observed. The same observations were noted in other representatives of ruminants like cattle and European elk (König 1979; Zdun et al. 2013, 2019). The absence of this part of these vessels was also noted in some antelopes from Antilopinae, Tragelaphus, Taurotragus, and Boselaphus (Frąckowiak et al. 2014, 2015). In the above-mentioned papers, only adult specimens were analyzed.

The main source of blood to the rostral epidural rete mirabile were branches from the maxillary artery. The caudal branch to the rete mirabile was a single vessel. It was a relatively strong artery in goat and sheep (Atalgın et al. 2011; Graczyk and Zdun 2021), while in Bovini was weaker, and the smallest lumen was in cervids (Graczyk and Zdun 2021). The rostral branches to the rete mirabile were always multiple vessels. Such information was noted in several described ruminants. Usually, two to four vessels were present, but not all papers defined the number of vessels (Atalgın et al. 2011; Graczyk and Zdun 2021; König 1979; Nickel et al. 1976; Wang et al. 2012; Zdun et al. 2013; Zdun et al. 2019).

The intracranial part of the internal carotid artery formed the cerebral arterial circle. Only in the dromedary camels the rostral cerebral artery and the caudal communicating artery emerged independently from rete mirabile, not from the internal carotid artery (Al Aiyan et al. 2019;). In other representatives of Artiodactyls no such pattern has been observed. In the analyzed species, the rostral communicating artery was observed. In representatives of Bovini, goat, Eurasian elk, and fallow deer, in most described specimens, this artery is present (Brudnicki 2000, 2011; Zdun et al. 2013, 2019). The research conducted on cattle showed, that the presence of this artery was noted only in a few cases (König 1979). Another issue that differs between the described species is the point, where the rostral choroidal artery branched off. This study shows, that this vessel branched off from the rostral cerebral artery or from the caudal communicating artery. In most described species, the rostral choroidal artery branched off from the rostral cerebral artery (Brudnicki 2000, 2011; Frąckowiak et al. 2014, 2015; Zdun et al. 2013, 2019). The rostral choroidal artery branched off from the caudal communicating artery in tapir (Frąckowiak and Giejdasz 1998). The double caudal cerebral artery was noted in the European roe deer (Godynicki and Wiland 1971) and only in part of analyzed specimens as the vascular variation in cattle, banteng, European bison, Eurasian elk, nyalas, sitatungas, Antilopinae (Frąckowiak et al. 2014, 2015; Zdun et al. 2013, 2019).

The basilar artery is a vessel with a smaller diameter in the caudal part. Similar observations were made in other ruminants (Brudnicki 2000; Frąckowiak et al. 2014, 2015; Godynicki and Wiland 1971; Zdun et al. 2013, 2019). Baldwin and Bell (1960 and 1963) performed experiments on cattle and sheep. The Authors state, that the basilar artery does not participate in the supply of blood from the vertebral arteries to the brain. This vessel presents itself differently in the camel, in which it is not thinner in the caudal part, but is an important source of blood for the brain (Al Aiyan et al. 2021; Jerbi et al. 2019).

## Conclusions

In the article, we describe arterial vascularization of the brain of the Scimitar-horned oryx. We used three different anatomical methods for a complete image of the vascular pattern of the described area. The study’s results may contribute to advanced research in the fields of physiology or veterinary medicine.

## Data Availability

The datasets generated during and/or analysed during the current study are available from the corresponding author on reasonable request.
